# Denoising and Baseline Correction of Low-Scan FTIR Spectra: A Benchmark of Deep Learning Models Against Traditional Signal Processing

**DOI:** 10.3390/bioengineering13030347

**Published:** 2026-03-17

**Authors:** Azadeh Mokari, Shravan Raghunathan, Artem Shydliukh, Oleg Ryabchykov, Christoph Krafft, Thomas Bocklitz

**Affiliations:** 1 Leibniz Institute of Photonic Technology, Member of Leibniz Health Technologies, Member of the Leibniz Centre for Photonics in Infection Research (LPI), Albert‑Einstein‑Strasse 9, 07745 Jena, Germany; 2 Institute of Physical Chemistry (IPC) and Abbe Center of Photonics (ACP), Friedrich Schiller University Jena, Member of the Leibniz Centre for Photonics in Infection Research (LPI), Helmholtzweg 4, 07743 Jena, Germany

**Keywords:** Fourier Transform Infrared (FTIR) imaging, baseline correction, spectral denoising, physics-informed neural networks, cascade Unet

## Abstract

High-quality Fourier Transform Infrared (FTIR) imaging usually needs extensive signal averaging to reduce noise and drift, which severely limits clinical speed. Deep learning can accelerate imaging by reconstructing spectra from rapid, single-scan inputs. However, separating noise and baseline drift simultaneously without ground truth is an ill-posed inverse problem. Standard black-box architectures often rely on statistical approximations that introduce spectral hallucinations or fail to generalize to unstable atmospheric conditions. To solve these issues, we propose a physics-informed cascade Unet that separates denoising and baseline correction tasks using a new, deterministic Physics Bridge. This architecture forces the network to separate random noise from chemical signals using an embedded SNIP layer to enforce spectroscopic constraints instead of learning statistical approximations. We benchmarked this approach against a standard single Unet and a traditional Savitzky–Golay smoothing followed by SNIP baseline correction workflow. We used a dataset of human hypopharyngeal carcinoma cells (FaDu). The cascade model outperformed all other methods, achieving a 51.3% reduction in RMSE compared to raw single-scan inputs, surpassing both the single Unet (40.2%) and the traditional workflow (33.7%). Peak-aware metrics show that the cascade architecture eliminates spectral hallucinations found in standard deep learning. It also preserves peak intensity with much higher fidelity than traditional smoothing. These results show that the cascade Unet is a robust solution for diagnostic-grade FTIR imaging. It enables imaging speeds 32 times faster than current methods.

## 1. Introduction

Fourier Transform Infrared (FTIR) imaging generates label-free, high-resolution biochemical maps and is increasingly used to characterize cellular composition and metabolic heterogeneity in cancer models [1,2], including hypopharyngeal carcinoma (FaDu) cell lines. Beyond basic research, FTIR imaging has promising potential in high-throughput biomedical workflows because it yields intrinsic chemical contrast without staining or labeling [3,4]. However, broader practical use is constrained by a persistent trade-off between acquisition speed and spectral fidelity [3,5,6]. This limitation is especially severe for small and thin specimens such as single cells, which exhibit relatively weak absorption bands and therefore require high signal-to-noise ratios to preserve biochemically meaningful peak structure.

In standard protocols, spectra are averaged over many scans per pixel (e.g., 32 to 128) to suppress instrumental noise and atmospheric interference. While this produces high-quality (HQ) measurements, it makes imaging too slow for large fields of view and large tissue sections, thereby limiting practical throughput.

In contrast, 1 scan acquisition is fast but yields low-quality (LQ) spectra dominated by high-frequency noise and nonlinear baseline drifts caused by scattering effects and water vapor variability [7,8,9]. Here, scattering-related distortions, including possible Mie-scattering contributions, are treated as part of the broader baseline distortion problem rather than as a separately quantified variable. Consequently, enabling rapid FTIR imaging for biomedical use requires computational methods that can reliably recover HQ chemical signatures from noisy single-scan inputs.

Current restoration strategies can be divided into traditional signal processing pipelines and data-driven deep learning methods [8,10,11,12]. The standard chemometric workflow typically combines Savitzky–Golay (SG) smoothing for denoising [13,14,15] with the Sensitive Nonlinear Iterative Peak (SNIP) algorithm for baseline correction [16]. These approaches are interpretable and grounded in established preprocessing practice. However, they exhibit important limitations in high-throughput settings. SG filtering behaves as a low-pass operator and can systematically broaden peaks and reduce effective spectral resolution, potentially degrading peak-dependent biochemical interpretation [16,17,18]. In addition, SNIP is iterative and can introduce computational latency when applied at scale across dense hyperspectral images [16]. More importantly, when LQ inputs contain both strong stochastic noise and substantial baseline drift, a fixed preprocessing pipeline may struggle to suppress noise while preserving subtle chemical features.

Deep learning architectures, including Unet variants, offer a robust alternative because they can learn nonlinear mappings and perform rapid inference once trained [8,10,11,12]. Yet standard end-to-end neural networks lack explicit spectroscopic constraints. In real experimental data, separating random noise from background drift without direct access to the underlying clean spectrum is an ill-posed inverse problem. This ambiguity can force a purely data-driven model to rely on dataset-specific statistical shortcuts, which may lead to hallucinated spectral features or degraded performance under out-of-distribution acquisition conditions such as changes in atmospheric contributions [17]. These limitations motivate hybrid strategies that combine the expressive power of neural networks with explicit, physics-aligned preprocessing constraints.

To address this gap, we propose a physics-informed cascade Unet that integrates the robustness of physical preprocessing with the flexibility of deep learning. Unlike end-to-end architectures that attempt to learn denoising and baseline correction in a single step, our approach decomposes restoration into two stages linked by a physics-informed bridge. Specifically, the bridge integrates a deterministic, non-learnable SNIP layer into the forward pass, ensuring that baseline suppression is performed using established spectroscopic principles. By transferring baseline modeling to the SNIP operator, the learnable components are freed to focus on high-frequency denoising and fine-scale artifact refinement, improving interpretability and reducing the risk of physically implausible reconstructions.

In this work, we make three main contributions. First, we introduce a two-stage cascade architecture that explicitly separates denoising and baseline refinement, reducing ambiguity compared with single-stage end-to-end learning. Second, we incorporate a deterministic Physics Bridge by embedding SNIP within the inference path, thereby anchoring restoration to established baseline correction principles and improving robustness under real acquisition variability. Third, we provide a rigorous benchmark on biological FTIR imaging data using leave-one-sample-out evaluation together with both global fidelity metrics and peak-aware chemical fidelity metrics, comparing against a standard single Unet and a tuned traditional SG + SNIP workflow.

Overall, this framework supports improved peak integrity and stability, suggesting a practical pathway toward reliable interpretation from extremely low-scan acquisitions (e.g., single-scan) and thereby enabling higher-throughput FTIR imaging for biomedical applications.

## 2. Materials and Methods

### 2.1. Dataset Description

To evaluate the proposed denoising architectures on complex biological data, we utilized FTIR spectroscopic images derived from the FaDu cells cultivated on 1 mm-thick CaF_2_ windows. FaDu refers to a human hypopharyngeal squamous cell carcinoma cell line commonly used in head and neck oncology research. The FaDu cell line was obtained from DSMZ (German Collection of Microorganisms and Cell Cultures, Braunschweig, Germany; catalog no. ACC 784; RRID: CVCL_1218). These samples were selected to ensure the model encounters realistic biochemical heterogeneity, as varying cell densities and metabolic activities within the samples produce diverse spectral signatures rather than uniform synthetic signals.

Data were acquired with the FTIR spectrometer 670 and the microscope 620 (Agilent Technologies, Santa Clara, CA, USA) equipped with a liquid-nitrogen-cooled 64 × 64 focal plane array (FPA) detector. Acquisition was performed in transmission mode using a 15× Cassegrain condenser and objective lenses as previously described [18] on four distinct sample regions, designated as FaDu1 through FaDu4. These independent fields of view (FOVs) provide the variance needed for robust cross-validation. This ensures the model generalizes across different biological structures. Notably, the dataset includes real-world experimental variability; for instance, the FaDu3 sample shows spectral drift due to environmental instability (e.g., humidity or purge gas fluctuations). This provides a rigorous test for denoising stability in non-ideal conditions.

To construct a supervised learning benchmark, it is essential to have paired noisy and clean representations of the exact same biological target. Consequently, each FOV was imaged under three distinct signal accumulation settings.

The LQ inputs were acquired using 1 scan and 8 scans per pixel. These modes simulate high-speed imaging scenarios where acquisition speed is prioritized, resulting in data characterized by lower signal-to-noise ratios (SNR) and significant instrumental noise contributions.

The HQ ground truth was acquired using 32 scans per pixel. The 32-scan spectra possess a statistically superior SNR and serve as the high-quality ground truth for model training and quantitative evaluation.

**Figure 1:**
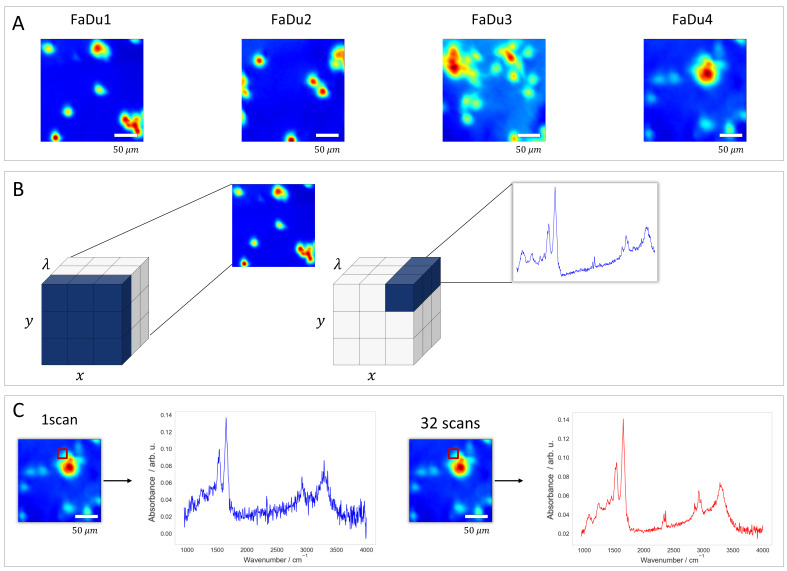
(**A**) Chemical images displaying the four distinct sample regions (FaDu1–FaDu4). (**B**) Illustration of the hyperspectral data cube format (x, y, λ). The schematic demonstrates how a slice along the λ-axis represents a spatial map, while each individual spatial pixel corresponds to a full infrared absorbance spectrum containing the sample’s biochemical fingerprint. (**C**) Comparison of spectral quality based on scan accumulation. The panels show spectra extracted from the same spatial pixel (indicated by the red square) using 1 scan versus 32 scans, highlighting the improvement in signal-to-noise ratio with increased scanning. All FTIR image panels are 64 × 64 pixels and were acquired with the same 15× objective, corresponding to a field of view of approximately 350 × 350 µm (5.5 µm per pixel). Therefore, all images are shown at the same spatial scale. Differences between the 1-scan and 32-scan images reflect signal quality rather than spatial extent. The scale bar shown applies to all FTIR image panels, which were acquired at the same spatial scale.

The resulting hyperspectral data cubes are three-dimensional tensors. Each image has spatial dimensions of 64 × 64 pixels. The spectral dimension consists of 1584 data points that covers the mid-infrared fingerprint and functional group regions (approximately 950 to 4000 cm^−1^, with a spectral resolution of 4 cm^−1^ and a data interval of roughly 2 cm^−1^ at interleave factor 1). This high-dimensional structure forces the algorithms to handle both spatial correlations between pixels and complex spectral features in biological tissues. A visual comparison of the signal quality across the different scan accumulations is presented in Figure 1.

### 2.2. Dataset Preparation and Preprocessing

The intrinsic complexity of FTIR spectra interpretation is frequently impeded by the existence of extraneous factors and measurement variability. Variations occur over time within the same sample or between biological replicates. Common causes include biological drift, sample deterioration, inconsistent preparation, and other uncontrolled experimental factors [19,20]. Such sources of variation, if not addressed, can obscure meaningful spectral patterns and compromise the reliability of downstream analyses [21,22].

**Figure 2:**
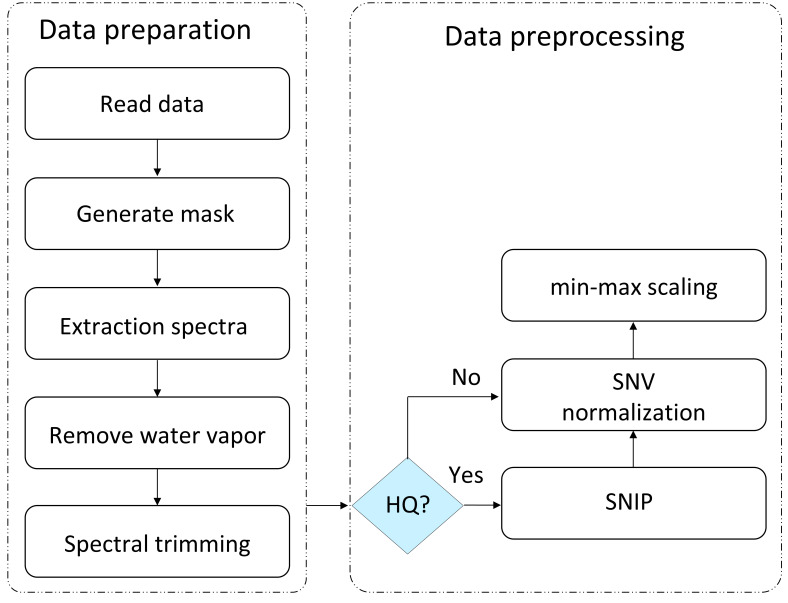
Schematic of the computational workflow illustrating the separation between data preparation and data preprocessing. The preparation phase isolates valid biological spectra through masking, water vapor correction, and spectral trimming. The preprocessing phase applies SNIP only to the HQ ground truth, while LQ inputs retain their baselines, before both undergo identical SNV and min–max normalization.

To address these issues, the raw hyperspectral data cubes underwent a comprehensive two-phase processing pipeline. This pipeline was designed first to isolate valid biological signals from physical artifacts (data preparation), and second, to standardize signal distributions for optimal neural network convergence (data preprocessing) as illustrated in Figure 2.

#### 2.2.1. Data Preparation

The initial phase focused on extracting cellular spectra from the hyperspectral image cubes. To distinguish biologically relevant information from the empty substrate, a binary mask was generated using Otsu’s thresholding method [23]. This thresholding was applied to the integrated intensity of the fingerprint region and the C-H stretching bond region. Consequently, only foreground pixels corresponding to cellular material were extracted for analysis, reducing computational overhead from non-informative background data.

Atmospheric fluctuations during scanning often introduce artifacts. To remove these environmental interferences, specifically water vapor contributions, an average background spectrum was computed from the non-sample pixels and subtracted from each foreground spectrum [24].

Finally, to eliminate regions containing no biological information, the spectral axis was pruned. This involved trimming the noisy detector edges and removing the 2250–2401 cm^−1^ region, which contains the strong atmospheric CO_2_ absorption band.

#### 2.2.2. Data Preprocessing

Following extraction, the workflow applied differential processing to define the supervised learning task. The goal is to train the model to map raw, distorted inputs to pure-absorbance targets. For the HQ target data (32 scans), the SNIP algorithm was applied immediately to remove the baseline, ensuring the network trained against pure absorbance targets with no baseline offsets. In contrast, the LQ (1 and 8 scans) input spectra bypassed the baseline removal step. Retaining the native baselines forces the model to learn the complex task of simultaneously suppressing the background and reducing random noise.

Following this split, we applied a two-step normalization process to both datasets to ensure numerical stability. First, Standard Normal Variate (SNV) normalization [25] was applied individually to each spectrum to reduce physical scattering effects. Second, a global min–max scaling was performed to map all spectral intensities into a stable [0, 1] range based on the global minimum and maximum values of the dataset.

### 2.3. Architectures Implemented

#### 2.3.1. Method a: Traditional Signal Processing (Benchmark)

To establish a rigorous baseline for evaluating deep learning models, we implemented a classic chemometric workflow that sequentially addresses the two main sources of spectral corruption: high-frequency instrumental noise and low-frequency baseline drift. This benchmark combines SG smoothing with the physics-based SNIP algorithm. In contrast to the proposed neural networks, this method relies entirely on deterministic signal-processing operations rather than learned parameters.

The initial processing stage addressed high-frequency instrumental noise within the LQ input spectra using a Savitzky–Golay filter. Because the dataset contains paired acquisitions of the same spatial pixels at LQ input (1 and 8 scans) and HQ ground truth (32 scans), the SG filter could be tuned in a supervised manner rather than by using default parameters. This is important because the SG response depends strongly on the choice of window length and polynomial order. Too little smoothing leaves substantial instrumental noise, whereas too much smoothing attenuates narrow absorbance bands and alters peak shape [14].

To eliminate arbitrary manual tuning and ensure the benchmark represented the theoretical limit of traditional processing, we utilized the Optuna framework to perform an automated Bayesian hyperparameter search [26]. Using the Tree-structured Parzen Estimator (TPE) algorithm, we optimized the filter’s window length and polynomial order. The optimization procedure is described in Section 2.4.2.

A key challenge in this workflow is the domain incompatibility between denoising and baseline correction. While denoising was optimized in the normalized [0, 1] domain to maintain metric consistency with the neural networks, the subsequent baseline correction algorithm requires physical intensity values to function correctly. The SNIP algorithm operates on geometric principles, specifically the iterative comparison of local intensity minima, which depends on the relative amplitude ratios of the original absorbance data. Normalization distorts these ratios, making geometric clipping ineffective.

To address this, we introduced a distinct architectural component termed the “Physics Bridge”. Mathematically, this is an inverse normalization (reversing the global min–max scaling and SNV normalization). However, it serves a critical function and a necessary transition from the statistical feature space (optimized for gradient descent and filtering) to the physical signal space (absorbance). This restoration ensures that the subsequent baseline correction is driven by spectroscopic constraints rather than statistical artifacts. Once restored to the physical domain, the spectra were processed using the SNIP algorithm to estimate and subtract the background continuum, effectively treating the broad underlying baseline as a low-frequency carrier wave to be isolated from the high-frequency chemical signal.

#### 2.3.2. Method b: Single Unet

To represent current data-driven methods, we implemented a standard single Unet architecture as an end-to-end regression model [27]. Unlike the traditional workflow, which decouples signal corruption into distinct frequency domains (treating noise and baseline as separate problems), this architecture assumes it can learn a direct nonlinear mapping from the raw LQ input to the corrected HQ target. This makes optimization difficult. The network must learn to suppress high-frequency stochastic noise while simultaneously identifying and subtracting low-frequency baseline drifts solely based on learned statistical correlations from the training examples.

Structurally, the model is designed as a 1D convolutional encoder–decoder network with mechanisms to improve feature selectivity. The encoder path utilizes successive convolutional blocks to downsample the input, extracting high-level latent features essential for distinguishing signal from noise. To mitigate the vanishing gradient problem and improve information flow through the deep network, the bottleneck section incorporates residual (ResNet) blocks [28]. Crucially, the decoder path is reinforced with spectral attention modules, which allow the network to dynamically recalibrate feature weights, thereby focusing capacity on chemically relevant spectral bands while suppressing irrelevant background signals.

To preserve high-frequency spatial information lost during downsampling, the network employs skip connections that concatenate features from the encoder directly to the decoder. In the context of vibrational spectroscopy, these connections are critical for retaining the structural integrity of sharp absorbance peaks that might otherwise be eroded by pooling operations. The model was trained in a fully supervised manner, mapping normalized LQ spectra (containing native baselines) directly to normalized, SNIP-corrected HQ targets. However, this black box approach operates without explicit physical constraints. By forcing the model to statistically approximate the geometric process of baseline removal, it becomes susceptible to spectral hallucinations—the generation of artifacts that mimic chemical features—particularly when inferring samples with environmental conditions that deviate from the training distribution.

#### 2.3.3. Method c: Cascade Unet (Physics-Informed Architecture)

To overcome the limitations of both rigid signal processing and black-box deep learning, we developed the physics-informed cascade Unet. This hybrid architecture separates spectral restoration task into two learnable phases, denoising and refinement, separated by a deterministic Physics Bridge. Unlike the single Unet, which must approximate the complex transformation from noisy, tilted inputs to flat, clean outputs in a single pass, the cascade model distributes these tasks to specialize the network components.

The first stage comprises a Unet trained strictly for noise suppression. Its input is the normalized LQ spectrum, containing both high-frequency noise and variable baseline drift. The target of optimization for this stage is the HQ spectrum with the baseline retained. This target definition prevents the network from having to distinguish low-frequency background drift from broad chemical features. This ambiguity is a primary source of hallucination in standard models. Consequently, Unet 1 acts as a high-fidelity denoiser. It learns to suppress stochastic noise while preserving the underlying spectral topology and background curvature.

The Physics Bridge sits between the two neural stages. It is a non-learnable computational layer that enforces spectroscopic constraints. The SNIP algorithm relies on geometric peak clipping that depends on absolute intensity ratios. Therefore, it does not function correctly in the normalized [0, 1] feature space. To address this, the bridge performs a differentiable inversion within the model graph. The output of Unet 1 is first mathematically inverted (inverse min–max followed by inverse SNV) using the input’s stored statistics to restore physical absorbance units. Subsequently, the restored spectra are processed by a TensorFlow-wrapped SNIP layer. This ensures that background removal follows established physical laws rather than statistical approximations. It provides stability against environmental drifts that typically confuse standard neural networks.

**Figure 3:**
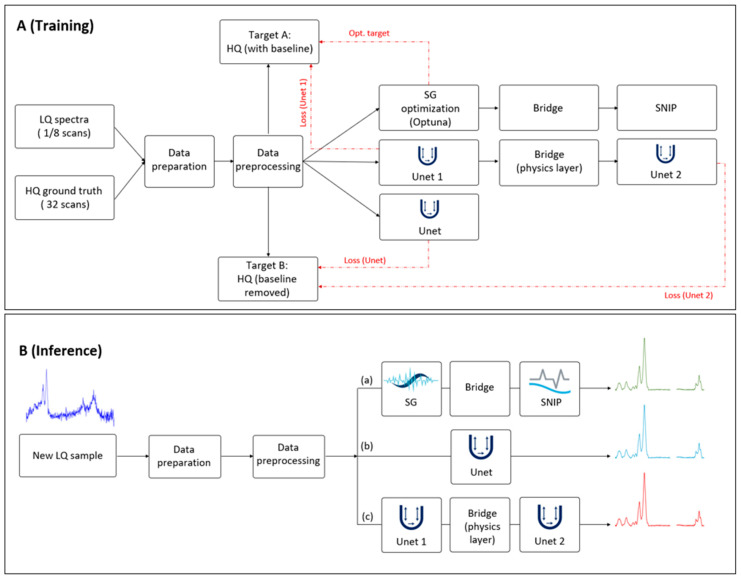
Computational workflow comparing multi-objective training and feed-forward inference. (**A**) Training phase: the diagram shows the parallel implementation of the three benchmarking methods. LQ inputs (1 & 8 scans) and HQ ground truth (32 scans) undergo identical data preparation and preprocessing. The proposed cascade Unet uses a decoupled optimization strategy. Unet 1 minimizes ${Loss}_{stage1}$ against Target A (HQ spectra with native baseline), focusing on denoising. The output is then processed by the Physics Bridge, which performs domain inversion and deterministic SNIP baseline removal, before passing to Unet 2, which minimizes ${Loss}_{stage2}$ against Target B (baseline-free HQ spectra). Red dashed lines indicate gradient and loss calculations. (**B**) Inference phase: the deployment workflow for processing unseen clinical samples. Optimization pathways and ground truth targets are deactivated. The model operates as a strictly feed-forward pipeline where the Physics Bridge remains active, ensuring baseline removal follows geometric spectroscopic constraints rather than learned approximations.

The baseline-free spectra emerging from the bridge are re-normalized and passed to the second stage, Unet 2. While SNIP is robust, it remains a mathematical approximation that can introduce systematic artifacts, such as the attenuation of broad peak bases or residual curvature. Unet 2 acts as a refinement network trained against the final baseline-free HQ ground truth. It learns a residual correction function to repair SNIP artifacts and recover high-resolution peak shapes. The architecture is trained using a deep supervision strategy with a multi-term loss function, computing the MSE at two distinct exit points: ${Loss}_{stage1}$ compares the output of Unet 1 (denoised + baseline) against the HQ target, while ${Loss}_{stage2}$ compares the final output of Unet 2 against the baseline-free HQ target. The complete workflow, contrasting the multi-objective training path with the streamlined inference pipeline used for processing new clinical samples, is illustrated in Figure 3.

During the inference (test) phase, the optimization pathways, including the HQ targets and loss calculation blocks shown in Figure 3A, are deactivated. The deployed model operates as a feed-forward pipeline (Figure 3B); a new LQ spectrum is denoised by Unet 1, processed through the deterministic Physics Bridge to remove the baseline, and finally refined by Unet 2. Unlike standard domain adaptation techniques that might be removed after training, the Physics Bridge remains an active, integral component of the inference graph. This ensures that every prediction, whether in training or clinical deployment, adheres to the same spectroscopic constraints.

### 2.4. Experimental Design

#### 2.4.1. Cross-Validation Strategy

To ensure the clinical applicability and robustness of our models, we used a strict Leave-one-sample-out cross-validation (LOSO-CV) strategy [29]. The dataset was partitioned by biological sample, corresponding to the four independent fields of view labeled FaDu 1 through FaDu 4. In each validation iteration, models were trained on spectra from three sample regions and evaluated exclusively on the held-out fourth sample. This design was chosen specifically to prevent data leakage, ensuring that reported metrics reflect the model’s ability to generalize to unseen biological heterogeneity rather than its capacity to memorize sample-specific spectral distributions.

#### 2.4.2. Hyperparameter Optimization

For the traditional benchmark (Method a), the SG filter parameters were optimized using Optuna with the Tree-structured Parzen Estimator. The search was performed within the same LOSO-CV framework used for model evaluation, where the four independent FaDu regions served as the held-out fields of view. In each LOSO iteration, one FOV was held out for evaluation, and all preprocessing parameters for that iteration were derived only from the remaining three FOVs.

After spectral trimming and removal of the 2250–2401 cm⁻¹ CO₂ region, each spectrum was first transformed by SNV normalization. A train-only min–max range was then estimated and used to scale both the corresponding training data and the held-out data into the normalized domain. SG smoothing was applied only to the LQ (1 scan and 8 scans) spectra, while the matched 32-scan spectra were kept unchanged as the reference target.

The search space included odd window lengths from 5 to 61 points and polynomial orders from 2 to 5, with the derivative order fixed at zero so that the filter was used only for smoothing. Optuna was run for 60 trials.

For each candidate parameter set, performance was evaluated across all four LOSO held-out FOVs. In each held-out FOV, the SG-smoothed LQ spectra were compared with the matched HQ spectra in the normalized domain using a composite objective based primarily on MSE, with a small additional SAM penalty to discourage spectral shape distortion. The final SG parameters were selected as the combination that minimized this mean cross-validated objective across the four held-out FOVs.

#### 2.4.3. Neural Network Training Protocol

Both deep learning architectures, the single Unet and cascade Unet, were implemented in TensorFlow and trained using identical hyperparameters to isolate the impact of architectural differences. Training utilized the Adadelta optimizer with a learning rate of 0.05 and a batch size of 32.

To reduce overfitting and avoid unnecessary training once validation performance had stabilized, an early stopping strategy was applied based on the validation loss. The patience was set to 30 epochs, which was selected empirically during preliminary experiments as a practical compromise between allowing for short-term validation fluctuations and preventing premature stopping. Models were trained for a maximum of 5000 epochs, and the weights corresponding to the best validation performance were restored for final inference. The same training protocol was used across all LOSO iterations and model variants to ensure a fair comparison.

#### 2.4.4. Evaluation Framework

Model performance was assessed using complementary metrics designed to capture both statistical reconstruction accuracy and chemical validity. To ensure a fair comparison across methods, all metrics were computed in a common evaluation domain by comparing outputs to the normalized, baseline-free HQ ground truth (stage-2 min–max space). The processed input baseline (LQ) was subjected to the same baseline removal and normalization steps so that it was evaluated in the same domain as the HQ target.

Let $y^{\left(i\right)}\in R^{L}$ denote the HQ target spectrum and ${\hat{y}}^{\left(i\right)}\in R^{L}$ the reconstructed spectrum for spectrum i, with L spectral samples. To quantify overall reconstruction fidelity, we report RMSE and MAE:

$${RMSE}^{\left(i\right)}=\sqrt{\frac{1}{L}\sum_{j=1}^{L}{\left({\hat{y}}_{j}^{\left(i\right)}|-|y_{j}^{\left(i\right)}\right)}^{2}}$$

$${MAE}^{\left(i\right)}=\frac{1}{L}\;\sum_{j=1}^{L}\left|{\hat{y}}_{j}^{\left(i\right)}-y_{j}^{\left(i\right)}\right|$$

Because pointwise errors may not fully reflect spectral shape preservation, we additionally compute the Spectral Angle Mapper (SAM) alongside the Pearson Correlation Coefficient (PCC). SAM was reported in degrees:

$${SAM}^{\left(i\right)}=\;\;\frac{180}{\pi }\arccos\left(\frac{{\hat{y}}^{\left(i\right)}\;.\;y^{\left(i\right)}}{{\left\|{\hat{y}}^{\left(i\right)}\right\|}_{2}\;\;{\left\|y^{\left(i\right)}\right\|}_{2}}\right)$$

$${PCC}^{\left(i\right)}=\frac{\sum_{j=1}^{L}\left({\hat{y}}_{j}^{\left(i\right)}-{\bar{\hat{y}}}^{\left(i\right)}\right)\left(y_{j}^{\left(i\right)}|-|{\bar{y}}^{\left(i\right)}\right)}{\sqrt{\sum_{j=1}^{L}{\left({\hat{y}}_{j}^{\left(i\right)}|-|{\bar{\hat{y}}}^{\left(i\right)}\right)}^{2}}\;\;\sqrt{\sum_{j=1}^{L}{\left(y_{j}^{\left(i\right)}|-|{\bar{y}}^{\left(i\right)}\right)}^{2}}}$$

where ${\bar{\hat{y}}}^{\left(i\right)}$ and ${\bar{y}}^{\left(i\right)}$ denote the mean intensity values of the reconstructed and target spectra, respectively.

To complement global error metrics, we evaluated peak preservation using peak-aware chemical fidelity measures. Peaks were detected in both the HQ target and reconstructed spectra using the same prominence criterion in the normalized domain (prominence threshold =0.02). To reduce spurious detections, at most 20 peaks per spectrum were retained, ranked by prominence. Peaks were then matched by nearest-neighbor association within a tolerance of ±4 spectral samples. Matching was performed in spectral index space, while the reported peak position error was expressed in wavenumber units (cm^−1^) using the spectral sampling interval.

For each matched pair with target peak index $v_{t}$ and predicted peak index $v_{p}$ the absolute peak position error was computed as:

$$Epos=|\nu (\nu_{p})-\nu (\nu_{t})|$$

where $\nu \left(\cdot \right)$ maps spectral index to wavenumber. Peak amplitude preservation was quantified using the peak height bias evaluated at the matched maxima:

$$E_{amp}=\hat{y}(\nu_{p})-y(\nu_{t})$$

For each method and LOSO iteration, all metrics were first computed per spectrum against the normalized, baseline-free HQ reference. The resulting per-spectrum values within the held-out FOV were then summarized by the median. Final reported values correspond to the mean ± standard deviation across the four held-out FOV summaries.

For Table 1, the percentage reduction for RMSE, MAE, and SAM relative to the processed input baseline was calculated as:

$$Reduction(\%)=100\times \frac{M_{LQ}-M_{method}}{M_{LQ}}$$

where $M_{LQ}$ is the metric value for the processed input baseline and $M_{method}$ is the corresponding value for the evaluated reconstruction method.

To provide a targeted qualitative comparison on difficult examples, representative LQ spectra were selected for visualization using a heuristic combined-ranking strategy. First, for each LQ spectrum, we computed the per-spectrum RMSE and SAM relative to the normalized HQ reference. In addition, two simple signal quality proxies were computed from the raw physical LQ spectra: a noise proxy, defined as the standard deviation of the residual after SG smoothing, and a baseline distortion proxy, defined as the fraction of low-frequency spectral power estimated in the Fourier domain. Each quantity was standardized using a z-score, and a combined difficulty score was then defined as

$$S^{\left(i\right)}=z({RMSE}^{\left(i\right)})+z({SAM}^{\left(i\right)})+z({Noise}^{\left(i\right)})+z({Baseline}^{\left(i\right)}).$$

Spectra with larger $S^{\left(i\right)}$ values were interpreted as more challenging because they simultaneously exhibited greater deviation from the HQ reference, stronger shape distortion, higher residual noise, and more pronounced broad baseline or scattering-related structure. The final spectra used for qualitative visualization were chosen from the highest-ranked candidates after manual inspection to ensure visually distinct and representative examples. This ranking was used only for figure selection and did not affect model training, parameter tuning, or quantitative performance evaluation.

For the derivative-based qualitative analysis , second derivatives were computed for the same selected spectrum using a Savitzky–Golay second-derivative filter (derivative order = 2, window length = 17, polynomial order = 3) with respect to wavenumber, using the same common normalized evaluation domain as used for the qualitative spectral comparison. 

## 3. Results

### 3.1. Quantitative Performance (Global Metrics)

The quantitative evaluation of spectral reconstruction accuracy reveals a distinct performance hierarchy among the tested architectures, with the physics-informed approach demonstrating superior fidelity across all global metrics. Metrics were first computed per spectrum against the normalized, baseline-free HQ reference, then summarized by the median within each held-out FOV in the LOSO-CV framework and finally reported across the four held-out FOVs as mean ± standard deviation. To establish a standardized baseline for improvement, we evaluated the processed input (LQ) baseline in the same normalized, baseline-free domain as the reconstructed outputs and the HQ reference. For Table 1, percentage reduction was calculated relative to the processed input (LQ) baseline for each metric as described in Section 2.4.4. As summarized in Table 1, the traditional SG + SNIP workflow achieved a baseline reduction in RMSE of 33.67% compared to the raw input. The standard single Unet improved upon this benchmark, yielding a 40.23% reduction in RMSE, validating the capability of deep learning to extract signal from noise more effectively than fixed mathematical filters. However, the proposed cascade Unet significantly outperformed both comparators, achieving an RMSE reduction of 51.30%. This trend was consistent across MAE metric, where the cascade model demonstrated a 52.13% error reduction, compared to 42.34% for the single Unet and 34.66% for the traditional workflow.

**Figure 4:**
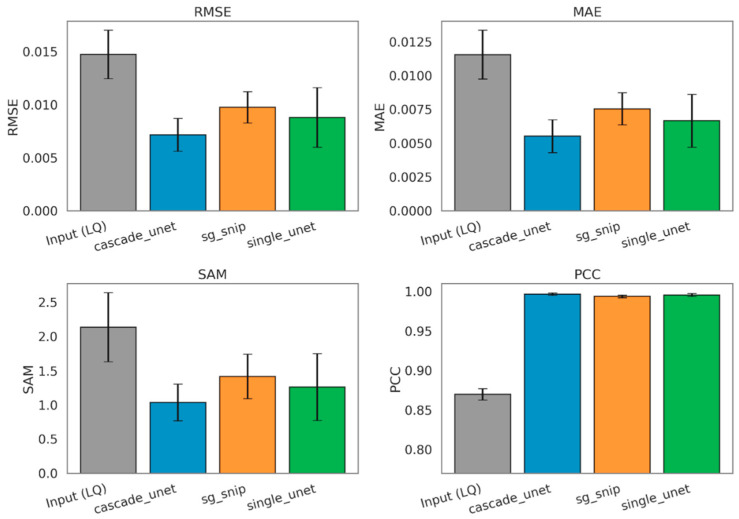
Comparative analysis of global spectral reconstruction fidelity across benchmarking methods. The bar charts display the mean RMSE, MAE, SAM, and PCC values obtained after computing each metric per spectrum against the normalized, baseline-free HQ reference and summarizing values by the median within each held-out FOV. Final bars show the mean across the four held-out FOVs, and error bars represent the corresponding standard deviation across FOV-level summaries. Methods shown are the processed input (LQ) baseline (gray), the proposed cascade Unet (blue), the traditional SG + SNIP workflow (orange), and the standard single Unet (green). (**Top Row**) Intensity-based metrics: RMSE and MAE. Lower values indicate better performance. The cascade Unet achieves the lowest error in both categories. (**Bottom Row**) Shape-based metrics: SAM, where lower values indicate better vector alignment, and PCC, where values closer to 1.0 indicate higher shape similarity. The cascade Unet demonstrates superior preservation of spectral topology compared to both the traditional and single-stage deep learning benchmarks.

Beyond intensity reconstruction, we assessed the preservation of spectral shape using the SAM and PCC. The raw LQ spectra showed a baseline PCC of 0.87, indicating significant corruption of the biochemical fingerprint. While all three denoising methods successfully restored the correlation to above 0.99, distinguishing their performance required the more sensitive vector-based SAM metric. The cascade Unet reduced the spectral angle error by 51.52%, providing the closest vector alignment to the ground truth. In comparison, the single Unet and SG + SNIP methods achieved reductions of 40.97% and 33.71%, respectively. These results, visualized in Figure 4, confirm that the cascade architecture yields the most chemically accurate spectral shapes.

### 3.2. Stability and Robustness

While global averages demonstrate the superiority of the cascade architecture, an analysis of sample-specific performance reveals a critical distinction in model stability. Real-world spectroscopic data is frequently compromised by environmental instabilities, such as fluctuations in purge gas composition or humidity levels, which induce complex, nonlinear baseline drifts. We quantified acquisition stability using noise in a band-free background window, defined as the standard deviation of absorbance in the mean background spectrum, before trimming and other preprocessing steps, after subtracting a straight-line fit in the 2500–2600 cm⁻¹ region. Under predominantly random noise, scan averaging should reduce this metric approximately as $1/\sqrt{N}$ (expected ratios: 1 → 8 ≈ 2.8, 1 → 32 ≈ 5.7). FaDu3 deviated strongly from this behavior (1 → 8: 1.81, 1 → 32: 3.68) and retained the highest residual noise in this window at 8 and 32 scans (1.35 × 10^−4^ and 6.64 × 10^−5^), consistent with drift-like, non-averageable variability.

Consequently, this dataset served as a rigorous stress test for evaluating model robustness against non-ideal acquisition conditions.

**Figure 5:**
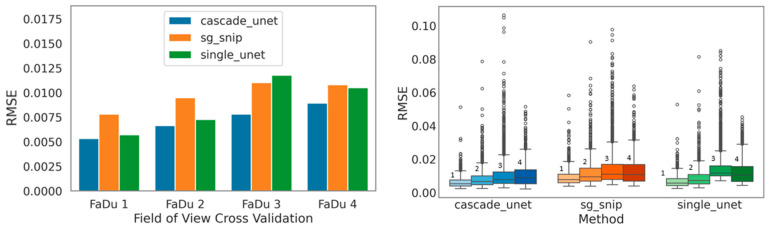
Assessment of model stability and generalization robustness across variable environmental conditions. (**Left**) RMSE stratified by LOSO-CV (FaDu 1–4). The cascade Unet (blue) maintains consistent low-error performance across all biological samples. In contrast, the single Unet (green) and traditional SG + SNIP (orange) exhibit notable performance degradation on the FaDu 3 dataset, which is characterized by significant environmental drift. (**Right**) Boxplots illustrating the distribution of reconstruction errors for each method. The single Unet displays a wider interquartile range and a high density of outliers, indicating instability and the generation of spectral artifacts. The cascade Unet achieves the most compact error distribution, confirming that the deterministic Physics Bridge effectively immunizes the model against baseline instabilities. It should be mentioned that within each method group, the labels 1, 2, 3, and 4 denote the corresponding LOSO-CV test sets FaDu 1, FaDu 2, FaDu 3, and FaDu 4, respectively.

Under these challenging conditions, the standard single Unet exhibited marked instability. As illustrated by the sample-wise RMSE boxplots (Figure 5), this architecture produced a wide distribution of errors, notably failing to maintain consistency on the drift-affected sample. This failure mode stems from the black-box nature of the architecture. Without explicit physical baseline knowledge, the model interpreted environmental drift as biological signal. This resulted in the generation of spectral hallucinations where the model effectively encoded the background noise into its prediction.

In contrast, the cascade Unet showed exceptional resilience to these environmental changes. By offloading the baseline removal task to the deterministic Physics Bridge (the embedded SNIP layer), the model became immune to background drift. The network did not have to learn the stochastic properties of environmental drift. This allowed the optimization to focus exclusively on signal restoration. This stability is visually confirmed by the plots in Figure 5, where the cascade model maintains a consistent, symmetric performance profile across all four biological samples. Conversely, the single Unet displays significant skewing and degradation in the presence of drift. The physics-informed approach thus delivers not only lower absolute error but also the reliability requisite for clinical pathology, where environmental conditions cannot always be perfectly rigorously controlled.

### 3.3. Peak Fidelity Analysis

Although global error metrics provide a macroscopic summary of reconstruction fidelity, they fail to explicitly quantify the preservation of diagnostic biochemical features. To validate the model’s utility for rigorous chemical analysis, we performed a peak-aware evaluation focusing on two critical parameters of infrared spectroscopy: peak localization and intensity conservation.

#### 3.3.1. Peak Position and Localization

**Figure 6:**
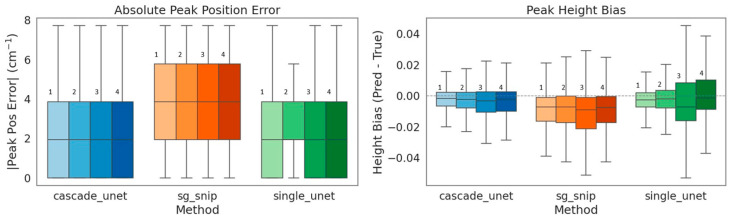
Evaluation of diagnostic chemical fidelity through peak-aware metrics. (**Left**) Absolute Peak Position Error: Boxplots show peak localization accuracy. The traditional SG + SNIP method (orange) has the largest deviations, attributable to phase shifts induced by polynomial smoothing. The deep learning models achieve tighter localization, with the cascade Unet (blue) minimizing spatial error. (**Right**) Peak Height Bias: Analysis of quantitative reconstruction accuracy. The dashed line at 0.0 represents zero bias (perfect reconstruction). The traditional method displays a systematic negative bias (median ≈ −1.6%), confirming the attenuation of peak intensity. While the single Unet (green) achieves a low median bias, it suffers from high variance (large interquartile range), indicating instability. The cascade Unet demonstrates superior precision (tightest IQR of 0.024) and minimal bias. It should be mentioned that within each method group, the labels 1, 2, 3, and 4 denote the corresponding LOSO-CV test sets FaDu 1, FaDu 2, FaDu 3, and FaDu 4, respectively.

Precise wavenumber localization is fundamental for the correct identification of specific molecular bonds. Absolute peak position error was calculated as the difference between the matched target and predicted peak locations and is reported in cm^−1^**.** Our analysis of absolute peak position error shows that the traditional SG + SNIP workflow produced the largest and most dispersed errors. This inaccuracy is largely attributable to the inherent limitations of polynomial smoothing, which effectively acts as a low-pass filter that can induce phase shifts in peak centers, particularly within asymmetric spectral bands as shown in Figure 6. In contrast, both deep learning architectures demonstrated superior localization accuracy. The cascade Unet achieved the tightest error distribution, indicating that the physics-informed model effectively distinguishes true signal maxima from noise without introducing the spatial distortions common to linear filters.

#### 3.3.2. Peak Intensity and Quantitative Bias

Since peak height serves as a direct proxy for molecular concentration, systematic bias in intensity reconstruction can lead to quantitative errors. Table 2 summarizes the peak height bias statistics, where bias was calculated as the signed amplitude difference (Pred − True) at the matched peak maxima, with negative values indicating underestimation of peak intensity. The traditional method showed a systematic negative bias, consistently underestimating peak heights by approximately 1.6%. This attenuation effect is characteristic of smoothing algorithms that average peak maxima with surrounding lower-intensity values.

The single Unet presented a contrasting failure mode; while it achieved a low median bias of −0.7%, it suffered from poor precision, evidenced by a high Interquartile Range (IQR) of 0.039. This high variance indicates that while the single Unet is accurate on average, its predictions fluctuate unpredictably for individual spectra, struggling to determine the correct amplitude in the presence of noise. The cascade Unet emerged as the most reliable estimator, combining the lowest bias (−0.6%) with the highest precision (IQR of 0.024). By removing the baseline via the Physics Bridge, the cascade network avoids the ambiguity of determining peak amplitude against a shifting background, resulting in tightly clustered and chemically reliable intensity predictions.

**Table 2:** Comparison of peak intensity reconstruction bias and precision. Peak height bias was computed per matched peak as the signed difference (Reconstructed − True) at the matched maxima. Bias is shown by the 25th, 25th (median), and 75th percentiles, while precision is represented by the Interquartile Range (IQR). The cascade Unet achieves the highest precision (lowest IQR of 0.024) and lowest median bias (−0.006 or −0.6%) compared to the traditional SG + SNIP method (median bias of −0.016 or −1.6%) and the single Unet method (highest IQR of 0.039 and median bias of −0.007 or −0.7%). The cascade’s superior performance is attributed to the effective baseline removal via the Physics Bridge.

Method	25th Perc	Median	75th Perc	IQR
**cascade**	−0.020	−0.006	0.005	0.024
**SG + SNIP**	−0.032	−0.016	−0.002	0.030
**single Unet**	−0.028	−0.007	0.011	0.039

### 3.4. Visual Inspection Outcomes

**Figure 7:**
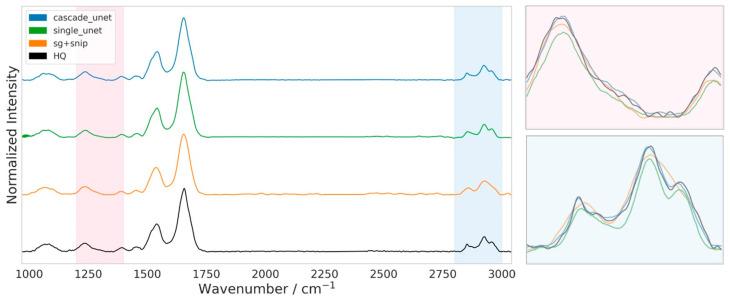
Qualitative comparison of spectral reconstruction fidelity in the fingerprint and high-wavenumber regions. Full-range stacked spectra (1000–3050 cm^−1^) for the cascade Unet (blue), single Unet (green), SG + SNIP (orange), and HQ ground truth (black). Shaded areas indicate the two evaluation windows: a representative fingerprint sub-region (pink, 1200–1400 cm^−1^) and the lipid-associated C-H stretching region (blue, 2800–3000 cm^−1^). Right panels show overlaid (no offset) zooms of the highlighted regions to enable direct comparison of peak shapes and amplitudes. SG + SNIP exhibits peak broadening and intensity attenuation, while the single Unet shows deviations in peak shape and amplitude stability. The cascade Unet most closely follows the HQ spectrum, preserving peak height, width, and fine shoulder structures.

To complement the statistical performance metrics, we conducted a qualitative visual assessment of the reconstructed spectra to verify the preservation of chemically diagnostic morphology. Figure 7 presents a representative spectral comparison between the three denoising architectures and the HQ ground truth. While all methods successfully recover the overall spectral shape across the fingerprint and functional group regions (1000–3000 cm^−1^), a closer examination of the high-frequency C-H stretching region (2800–3000 cm^−1^) reveals distinct differences in fine-structure preservation.

The traditional SG + SNIP workflow (orange trace) shows the typical limitations of polynomial smoothing filters. As evidenced in the zoomed inset, this method systematically erodes sharp spectral features, resulting in broadened peaks and a noticeable reduction in peak maxima relative to the HQ target. This flattening effect confirms the negative intensity bias quantified in the earlier peak-aware analysis, obscuring subtle shoulder features critical for lipid and protein discrimination.

The single Unet (green trace), while sharper than the traditional method, demonstrates a lack of localized precision. In the C-H stretching region, the model struggles to accurately resolve the valley depth between adjacent peaks and fails to match the absolute absorbance intensity of the ground truth. This visual deviation aligns with the higher interquartile range observed in the quantitative metrics, suggesting that the single-stage model has difficulty converging on the precise amplitude of complex multiplets.

In distinct contrast, the cascade Unet (blue trace) demonstrates a remarkable overlap with the HQ ground truth (black trace). The physics-informed architecture faithfully reproduces the intricate spectral topology, including the exact amplitude of peak maxima and the inflection points of the peak shoulders. By architecturally separating noise suppression from baseline management, the cascade model avoids both the over-smoothing of the Savitzky–Golay filter and the amplitude instability of the single Unet, yielding a reconstruction that is visually indistinguishable from the 32-scan reference.

**Figure 8:**
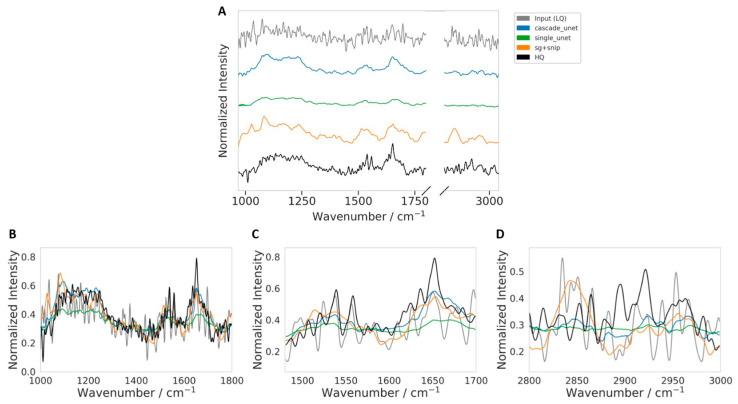
Qualitative comparison on a representative challenging LQ spectrum selected from the highest-ranked candidates according to the combined difficulty score described in Section 2.4.4. The selected spectrum exhibits pronounced noise together with broad scattering and baseline-related distortion. (**A**) Full spectral-range comparison (shown with vertical offsets for clarity) between the input (LQ), SG + SNIP, single Unet, cascade Unet, and HQ reference. (**B**) Fingerprint-region zoom. (**C**) Amide I/II-region zoom. (**D**) CHx-region zoom. All restoration methods improve substantially over the raw LQ input, while the cascade Unet more closely follows the HQ-like spectral profile across both the global spectrum and the key biochemical sub-regions. In contrast, the single Unet appears more attenuated in several regions, and SG + SNIP shows larger local amplitude deviations.

To complement the broader qualitative comparison shown in Figure 7, we examined one representative challenging LQ spectrum in greater detail. The spectrum shown in Figure 8 was selected for qualitative visualization using the combined difficulty-ranking procedure described in Section 2.4.4, based on per-spectrum RMSE, SAM, an estimate of residual noise (computed as the standard deviation of the residual after SG smoothing), and an estimate of low-frequency baseline and scattering distortion (computed from the fraction of low-frequency spectral power in the Fourier domain), followed by manual inspection of the highest-ranked candidates. We describe this example conservatively as a spectrum with pronounced noise and broad scattering and baseline-related distortion, rather than as part of a formally stratified high-Mie-scattering subset. Figure 8 shows the comparison over the full spectral range together with targeted views of the fingerprint, Amide I/II, and CHx regions.

Visual inspection of this difficult example confirms that the processed input LQ trace is substantially degraded by broad baseline and scattering-related distortion and local fluctuations, making direct biochemical interpretation unreliable. In the full-spectrum view (Figure 8A), all restoration methods suppress much of the non-chemical distortion and recover profiles that are visibly closer to the HQ reference. Differences between methods become clearer in the regional zooms. In the fingerprint region (Figure 8B), both the cascade Unet and SG + SNIP recover the main band structure more faithfully than the processed input LQ, whereas the single Unet appears more attenuated and locally flatter. In the Amide I/II region (Figure 8C), the cascade Unet more closely follows the broad envelope and dominant peak structure near the Amide I maximum, while the processed input LQ spectrum shows stronger oscillatory distortion and the single Unet underestimates the band amplitude. In the CHx region (Figure 8D), all restored spectra are smoother than the processed input, indicating effective suppression of spurious fluctuations. However, the cascade Unet appears to provide a better balance between noise reduction and preservation of the underlying regional trend, while SG + SNIP shows larger local amplitude deviations and the single Unet appears comparatively over-smoothed. Overall, Figure 8 provides a representative qualitative example in which the proposed cascade model better preserves HQ-like spectral structure under challenging distortion conditions.

**Figure 9:**
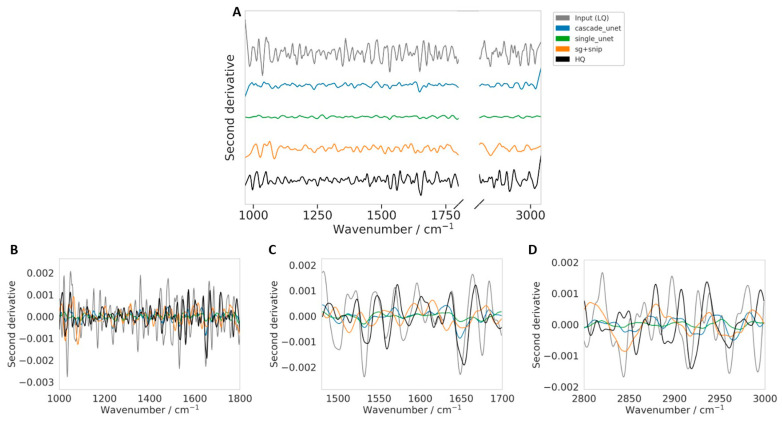
Second-derivative analysis of the representative challenging spectrum shown in Figure 8. The second derivative was computed for the processed input (LQ), SG + SNIP, single Unet, cascade Unet, and HQ reference using the same Savitzky–Golay differentiation settings for all spectra (second derivative, window length = 17, polynomial order = 3) to assess preservation of fine spectral structure relevant to derivative-based FTIR analysis. (**A**) Full spectral-range comparison (shown with vertical offsets for clarity). (**B**) Fingerprint-region zoom. (**C**) Amide I/II-region zoom. (**D**) CHx-region zoom. The processed input (LQ) remains highly unstable in the derivative domain, whereas all restoration methods reduce derivative noise substantially. Among the restored spectra, the cascade Unet more closely follows the HQ-like derivative pattern while remaining stable, whereas the single Unet appears more attenuated and SG + SNIP shows larger local deviations in some regions.

While the spectral overlays in Figure 8 provide a direct visual comparison in the absorbance domain, they do not fully reveal whether the restoration procedures preserve the finer band structure that is often exploited in derivative-based FTIR analysis. To address this point, we computed the second derivative spectra for the same challenging example and compared the processed input (LQ), SG + SNIP, single Unet, cascade Unet, and HQ reference in Figure 9. The second derivative was computed with respect to wavenumber using the same Savitzky–Golay differentiation settings for all spectra (window length = 17, polynomial order = 3, derivative order = 2). Because second derivatives enhance subtle band features while also amplifying residual noise, this representation provides a more stringent qualitative test of whether the denoising process preserves underlying biochemical information rather than only producing a visually smoother spectrum.

The second-derivative comparison in Figure 9 confirms that the processed input (LQ) remains highly unstable, with strong oscillations across the spectral range that obscure meaningful interpretation. All restoration approaches reduce this derivative instability substantially, indicating effective suppression of non-chemical fluctuations. However, the methods differ in how much derivative-level structure they preserve. The single Unet produces the flattest derivative profile, suggesting stronger attenuation of fine spectral information. In contrast, the cascade Unet retains more of the derivative-level pattern while maintaining a stable signal, particularly in the Amide I/II and CHx regions. The SG + SNIP workflow preserves stronger derivative variation than the single Unet but also exhibits larger local deviations from the HQ derivative shape in some regions. Overall, Figure 9 provides a representative qualitative example in which the proposed cascade model appears to better balance noise suppression with preservation of the underlying spectral structure relevant to downstream multivariate analysis.

### 3.5. Computational Efficiency

Deploying denoising models in clinical pathology depends on both reconstruction quality and computational efficiency. We evaluated the resource requirements for each method, quantifying learnable parameter counts, training duration, and inference latency per spectrum. As summarized in Table 3, the traditional SG + SNIP workflow operates with zero learnable parameters, as it relies on fixed mathematical kernels rather than weighted neural connections. Consequently, it requires negligible optimization overhead (0.01 h), limited only to the automated hyperparameter search (via Optuna) used to define the optimal window length and polynomial order.

However, this advantage disappears during inference phase. Despite lacking deep layers, the traditional method requires approximately 0.48 ± 0.02 ms to process a single spectrum. This latency is driven by the iterative nature of the SNIP algorithm and the polynomial calculations of the SG filter, which are typically executed sequentially on the CPU and do not benefit from the massive parallelization capabilities of modern GPU hardware.

In contrast, the deep learning models illustrate the distinct trade-offs inherent in data-driven approaches. The single Unet, comprising approximately 21.5 million parameters, required roughly 3.3 ± 0.3 h to train across the four validation fields of view. However, it emerged as the most efficient architecture for deployment, achieving an inference speed of just 0.28 ± 0.04 ms per spectrum. This performance, nearly twice the speed of the traditional benchmark, is directly attributable to the highly parallel nature of convolutional operations on the GPU, which allows for the simultaneous processing of large spectral batches.

The proposed cascade Unet, being a dual-stage architecture, represents the most computationally intensive model. It contains approximately 43 million parameters and required an average training duration of 14.8 ± 1.9 h. Its inference time increased to 2.29 ± 0.57 ms per spectrum. This increased latency is caused by the Physics Bridge bottleneck, where data must be transferred from the GPU to the CPU for the non-differentiable SNIP calculation and subsequently returned to the GPU for the second refinement stage. While significantly slower than the single Unet, this processing speed remains well within acceptable limits for high-throughput imaging. It translates to the processing of a typical 64 × 64 pixel image tile in under 10 s, a negligible duration compared to the substantial physical acquisition time saved by scanning at 1 scan rather than 32 scans.

## 4. Discussion

The superiority of the cascade Unet can be directly attributed to the structural integration of the Physics Bridge, which fundamentally alters the learning dynamics of the neural network. Standard deep learning models, such as the single Unet, are tasked with learning a complex, high-dimensional mapping that requires the simultaneous suppression of random noise and the estimation of variable background baselines. Our results from the drift-affected FaDu 3 dataset demonstrate the inherent risk of this coupled formulation; when the model encounters environmental conditions or baseline curvatures that deviate from the training distribution, it lacks the physical context to distinguish these anomalies from biological signals, leading to the generation of spectral hallucinations and false positive peaks.

The cascade architecture solves this by separating the optimization tasks. By defining the target for the first stage as the baseline-present spectrum, the first Unet is relieved of the ambiguity associated with background removal, allowing it to act as an efficient, nonlinear denoiser. The subsequent Physics Bridge, specifically the deterministic SNIP layer, acts as a non-learnable guardrail. Because SNIP operates on physical intensity values according to rigid geometric constraints, it is immune to the overfitting phenomena that plague learnable parameters. It reliably removes the baseline, even if the specific drift pattern was not in the training set. Consequently, the second stage receives a consistently flattened spectrum, requiring it to correct only minor mathematical artifacts rather than interpret complex environmental interferences. This architectural choice ensures that the model remains robust to the instrumental variations inevitable in clinical settings.

This study highlights the distinct trade-offs between data-driven and model-driven signal processing. The traditional Savitzky–Golay workflow represents a model-driven approach constrained by fixed mathematical assumptions. While robust and interpretable, it is mathematically rigid; it cannot differentiate between high-frequency noise and sharp chemical features if their frequency components overlap. This limitation was empirically verified by our peak fidelity analysis, where traditional processing systematically broadened peaks and underestimated intensities, effectively reducing the spectral resolution of the imaging system.

Conversely, the single Unet represents a pure data-driven approach. It offers exceptional flexibility and can learn to separate signal from noise in regimes where linear filters fail. However, this flexibility comes at the cost of stability; without physical constraints, the model is prone to overfitting noise patterns and memorizing baselines, leading to poor generalization on unseen or unstable data. The cascade Unet effectively synthesizes these approaches. It uses the learnable power of deep learning to perform superior denoising without resolution loss, while simultaneously anchoring the output to physical reality through the deterministic SNIP baseline correction. This hybrid approach delivers the precision of AI with the reliability of classical spectroscopy.

## 5. Conclusions

This study was motivated by the need to improve acquisition speed in FTIR imaging while preserving spectral fidelity. Standard FTIR protocols often require averaging many scans per pixel to achieve acceptable signal-to-noise ratios, which substantially limits throughput for large fields of view and mosaic-based imaging of extended biological samples. By showing that single-scan spectra can be computationally restored to closely approximate a 32-scan high-quality reference, our results support a substantial reduction in acquisition time and highlight the potential of physics-informed restoration for faster FTIR imaging workflows.

While the cascade Unet introduces additional computational cost relative to a standard single Unet, the measured inference time remains very small in the context of the overall imaging pipeline. In practice, the time saved by reducing physical scan duration is expected to outweigh the added computational overhead. Importantly, the improved preservation of peak structure, peak position, and intensity-related spectral features suggests that this gain in speed can be achieved without compromising the chemical information needed for downstream biomedical interpretation. Taken together, these findings support the proposed framework as a promising route toward higher-throughput FTIR imaging.

Despite these encouraging results, the present study has several limitations. First, the evaluation was performed on a relatively small dataset consisting of four independent FaDu fields of view acquired from a single biological model under one FTIR imaging configuration. Although this setting was appropriate for controlled benchmarking and included realistic acquisition variability, broader validation across additional cell lines, tissue sections, instruments, and laboratories will be necessary to establish generalizability more fully. Second, the 32-scan spectra used in this study serve as a practical high-quality experimental reference for supervised learning, but they should not be interpreted as an absolute physical ground truth under all acquisition conditions. Third, the benchmark comparison was limited to an optimized SG + SNIP workflow, and a standard single Unet, and future work should include comparison against a broader range of modern restoration and denoising approaches.

An additional limitation concerns scattering-related distortions, including possible Mie-scattering effects, which were not independently quantified as a separate evaluation variable in the present study. Accordingly, the present study does not provide a dedicated evaluation on spectra stratified by Mie-scattering severity, because scattering severity was not independently annotated in the available dataset. Instead, such effects were treated within the broader category of low-frequency baseline and physical scattering distortions present in the experimental LQ spectra. Within this scope, the proposed cascade framework is expected to remain beneficial because SNV normalization reduces multiplicative scattering contributions and the embedded SNIP-based Physics Bridge explicitly addresses broad baseline structure, thereby reducing the burden on the learned component. A targeted analysis of spectra stratified by scattering severity remains an important direction for future work. In addition, established Mie-scattering compensation methods could be used to generate corrected reference spectra for dedicated training/validation in a future, explicitly stratified study.

From an application perspective, future studies should evaluate whether the improved reconstruction fidelity demonstrated here translates into measurable gains in downstream biomedical tasks, such as digital pathology, tissue classification, tumor margin assessment, and quantitative biochemical mapping. Additional work is also warranted to test the method across multi-site and multi-instrument acquisition settings, and to further optimize the computational implementation of the Physics Bridge for large-scale hyperspectral imaging pipelines. Overall, the proposed method provides a strong foundation for future translational studies aimed at making FTIR imaging faster, more robust, and more practical for biomedical use.

## Figures and Tables

**Table 1:** Quantitative comparison of global spectral reconstruction fidelity. For each method, RMSE, MAE, and SAM were first computed per spectrum against the normalized, baseline-free HQ reference, summarized by the median within each held-out FOV, and then reported across the four held-out FOVs. The table shows the percentage reduction of each metric relative to the processed input (LQ) baseline. The cascade Unet outperformed the other methods across all metrics (RMSE, MAE, and SAM). It achieved reductions of 51.30%, 52.13%, and 51.52%, respectively, outperforming both the traditional SG + SNIP workflow and the standard single Unet. Bold values indicate the best performance in each category.

Method	RMSE Reduction (%)	MAE Reduction (%)	SAM Reduction (%)
**cascade**	**51.30**	**52.13**	**51.52**
**SG + SNIP**	33.67	34.66	33.71
**single Unet**	40.23	42.34	40.97

**Table 3:** Comparative analysis of computational resource requirements and inference latency. The table details the learnable parameter count, total training or optimization duration, and the mean inference time per spectrum for each denoising architecture. Training times represent the cumulative duration across LOSO-CV framework. Inference times are reported as mean ± standard deviation. The traditional SG + SNIP method utilizes zero learnable weights; the reported 0.01 h corresponds to the hyperparameter optimization phase (window length/polynomial order selection) via Optuna. While the single Unet offers the fastest inference speed due to GPU parallelization, the cascade Unet remains within clinically viable limits for high-throughput imaging despite the computational overhead of the Physics Bridge.

Method	Parameters	Training Time	Test Time
**cascade**	42,969,994	14.8 ± 1.9 h	2.29 ± 0.57 ms
**SG + SNIP**	0	0.01 h	0.48 ± 0.02 ms
**single Unet**	21,484,997	3.3 ± 0.3 h	0.28 ± 0.04 ms

## Data Availability

The data presented in this study are not publicly available due to privacy and ethical restrictions. However, the data may be made available from the corresponding author upon reasonable request and subject to ethical and institutional approval..
